# Medial Sural Artery Perforator Flap for Tongue and Oral Cavity Reconstruction With Native Tongue Tip Preservation: Report of Three Cases

**DOI:** 10.1002/micr.70196

**Published:** 2026-02-14

**Authors:** Gian Piero Mantovani, Samuele Baldelli, Claudio Gio Francesco Blessent, Massimo Pinelli, Francesco Mattioli

**Affiliations:** ^1^ Department of Plastic Surgery Policlinico di Modena, University of Modena and Reggio Emilia Modena Italy; ^2^ Department of Otolaryngology Head and Neck Surgery University Hospital of Modena Modena Italy; ^3^ Department of Plastic Surgery University of Modena and Reggio Emilia (Università di Modena e Reggio Emilia – UNIMORE) Modena Italy

**Keywords:** functional outcomes, medial sural artery perforator flap, oral cavity cancer, tongue reconstruction, tongue tip preservation

## Abstract

Compartmental hemiglossectomy for oral cavity squamous cell carcinoma creates composite tongue/floor defects in which balancing mobility, bulk, and a supple lining is challenging. We report three consecutive reconstructions using a medial sural artery perforator (MSAP) flap, emphasizing preservation of the uninvolved tongue tip left intentionally unattached to maximize residual mobility. A 28‐year‐old woman, a 50‐year‐old man, and a 36‐year‐old woman with lateral tongue SCC underwent compartmental resection with selective neck dissection; defects measured ~4 × 3, 5 × 4, and 6 × 5 cm. Thin fasciocutaneous MSAP flaps (5 × 4, 6 × 5, and 7 × 6 cm) were harvested from the medial calf and inset intraorally after tumor ablation, with end‐to‐end microvascular anastomoses to cervical recipient vessels (typically the lingual or superior thyroid artery and the external or internal jugular vein). All flaps survived without surgical complications; donor sites were closed primarily. Oral feeding resumed on postoperative day 13, 18, and 12, respectively; speech was comprehensible in all cases after standard rehabilitation. Follow‐up occurred 6 months after completion of adjuvant radiotherapy (case 1), during adjuvant radiotherapy (case 2), and 3 months after completion (case 3) with all patients tolerating a full oral diet of any consistency and calf scars were linear and inconspicuous with no lower‐limb deficits. These findings suggest that, for medium‐sized lateral tongue/floor defects, the MSAP provides a favorable balance of pliability and volume with low donor‐site morbidity; when oncologically feasible, preserving and not suturing the native tongue tip may further enhance mobility and functional recovery.

## 1 Introduction

Radical surgical margins remain one of the strongest independent prognostic factors for local control and survival in oral cavity squamous cell carcinoma (Bellini et al. 2025). In locally advanced tumors of the mobile tongue, several groups therefore advocate an en bloc resection of the primary tumor together with its anatomical pathways of spread (compartmental surgery), in order to achieve wide three‐dimensional margins and reduce the risk of residual microscopic disease. (Calabrese et al. 2009). These extended resections frequently result in large composite defects of the tongue and floor of mouth, for which microvascular free‐flap reconstruction is usually required to restore swallowing, speech, and oral competence (Zhang et al. 2024).

The radial forearm free flap (RFFF) and the anterolateral thigh (ALT) flap remain among the most commonly used options for tongue reconstruction because of their reliable anatomy, long pedicles and versatility in shaping different defect sizes. However, both are associated with relevant donor‐site morbidity: RFFF harvest leaves a conspicuous forearm scar, typically requires skin grafting and may cause sensory disturbance or weakness, while ALT flaps often leave a large thigh scar and can be excessively bulky for intraoral defects, with potential negative impact on articulation and oral containment. (Zhang et al. 2024; Dattilo et al. 2025). The medial sural artery perforator (MSAP) flap has recently gained attention as an alternative that may overcome some of these limitations (Chen et al. 2008; Titus et al. 2024; Hallock 2022; Al Omran et al. 2023; Toyserkani and Sørensen 2015; Sue et al. 2020; Banjongleelahong et al. 2024). t is a thin, pliable fasciocutaneous flap whose tissue characteristics resemble those of the RFFF while relocating the donor site to the medial calf, where primary closure usually results in a relatively small linear scar and limited functional impairment (Dattilo et al. 2025; Chen et al. 2008; Titus et al. 2024; Hallock 2022; Banjongleelahong et al. 2024). An expanding body of anatomical, clinical and meta‐analytic evidence supports the MSAP flap as a versatile option for head and neck reconstruction, including tongue defects, and positions it as an intermediate or “workhorse‐like” solution between the bulkier ALT and the thinner RFFF flaps (Dattilo et al. 2025; Titus et al. 2024; Hallock 2022; Al Omran et al. 2023; Toyserkani and Sørensen 2015; Sue et al. 2020; Banjongleelahong et al. 2024).

We present three consecutive cases of tongue and floor‐of‐mouth reconstruction using the free MSAP flap in patients with squamous cell carcinoma. In all cases, a key technical principle was preservation of the native tongue tip during compartmental resection and deliberate non‐suturing of the tip to the flap, in order to maximize residual tongue motility. This strategy was chosen to optimize functional outcomes, given the central role of the tongue tip in consonant production and overall speech intelligibility demonstrated by objective speech studies after glossectomy and flap reconstruction. (Sun et al. 2007; Matsui et al. 2007). The lingual tip can be preserved during compartmental surgery in cases where the tumor is located along the lateral margin and is sufficiently distant from the tip to allow wide‐margin resection while maintaining its preservation; this approach does not conflict with the principles of compartmental surgery, as all potential tumor spread pathways are nonetheless removed, and a wide anterior resection margin ensures adequate disease control at the level of the tip of tongue. It is established that tumors involving the tongue tip are associated with poorer speech outcomes (Matsui et al. 2007; Riemann et al. 2016), further underscoring the importance of preserving this structure whenever oncologically feasible. We report the technique and outcomes, emphasizing the MSAP flap's low donor‐site morbidity and its favorable intraoral contouring compared to conventional reconstructive options.

## 2 Case Reports

### 2.1 Surgical Technique

Across all three consecutive cases, the oncologic and reconstructive workflow was standardized. Each patient underwent compartmental hemiglossectomy with ipsilateral neck dissection, performed in parallel with flap harvest by a two‐team approach.

All patients underwent preoperative CT angiography (CTA) of both lower legs to assess the presence, course, and caliber of the medial sural artery (MSA). CTA allowed three‐dimensional visualization of the vascular anatomy, confirming that the MSA was present, of adequate caliber, and provided at least one dominant perforator supplying the medial aspect of the calf (Dusseldorp et al. 2014; He et al. 2014). Although rare, anatomical absence or hypoplasia of the MSA has been described in the literature and therefore needed to be excluded preoperatively (Titus et al. 2024; Hallock 2022). Bilateral imaging was performed to select the side with the most favorable anatomy for flap harvest. CTA also provided an overview of the number, caliber, depth and general intramuscular course of the perforators, and these findings were systematically correlated with a handheld Doppler examination performed preoperatively and again intraoperatively before flap elevation.

Preoperative markings were made by drawing a line between the midpoint of the popliteal crease and the medial malleolus. Perforators were most commonly found 8–12 cm distal to the popliteal crease along this line (Al Omran et al. 2023; Toyserkani and Sørensen 2015; Sue et al. 2020). The most reliable perforator was marked on the skin using the Doppler signal and was correlated with the CTA findings.

The patient was positioned supine with the leg externally rotated and flexed, providing good exposure of the medial calf and access to the popliteal fossa, while allowing a two‐team approach with simultaneous tumor ablation and flap harvest. A tourniquet was applied to the proximal thigh and inflated before incision to achieve a bloodless field.

The skin paddle was designed elliptically over the selected perforator, typically measuring 6–8 cm in length and 4–5 cm in width for tongue reconstruction—dimensions that consistently allowed primary closure of the donor site without the need for grafting. The initial incision was made along the anterior border of the skin paddle down to the deep fascia, which was elevated together with the skin and subcutaneous tissue. The fascia was anchored to the dermis with a few stay sutures to prevent shearing during flap elevation.

Subfascial dissection proceeded in an anteroposterior direction to identify the dominant perforator previously marked with the Doppler. Once the perforator was visualized, the surrounding field was widely undermined to expose its course while preserving the vessel and any adjacent perforators. In most cases, a single perforator provided sufficient perfusion for the skin paddle (Hallock 2022; Daar et al. 2019).

The perforator was then dissected intramuscularly through the gastrocnemius fibers. The fibers were gently split—not divided—following their natural orientation, as the MSA typically ran parallel to them. The pedicle lay at a relatively superficial depth (1–2 cm) within the muscle (Dusseldorp et al. 2014). Dissection continued proximally toward the popliteal fossa, clipping and dividing the collateral branches until the MSA and its two venae comitantes were fully skeletonized up to their origin from the popliteal vessels.

Once adequate pedicle length and vessel caliber (approximately 10–12 cm in length, 2–3 mm arterial diameter) were achieved, the tourniquet was released to assess perfusion and confirm flap viability. The posterior incision of the skin paddle was then completed, and the flap was fully elevated and isolated on its vascular pedicle.

The flap was subsequently transferred to the oral cavity and inset into the tongue and floor‐of‐mouth defect. Microvascular end‐to‐end anastomoses were performed to cervical recipient vessels—typically the lingual or superior thyroid artery and the external or internal jugular vein. The uninvolved native tongue tip was preserved and intentionally left unstitched to the flap edge to maximize residual mobility. All donor sites were closed primarily without the need for skin grafts, resulting in linear, well‐concealed scars on the medial calf.

### 2.2 Case 1

**FIGURE 1:**
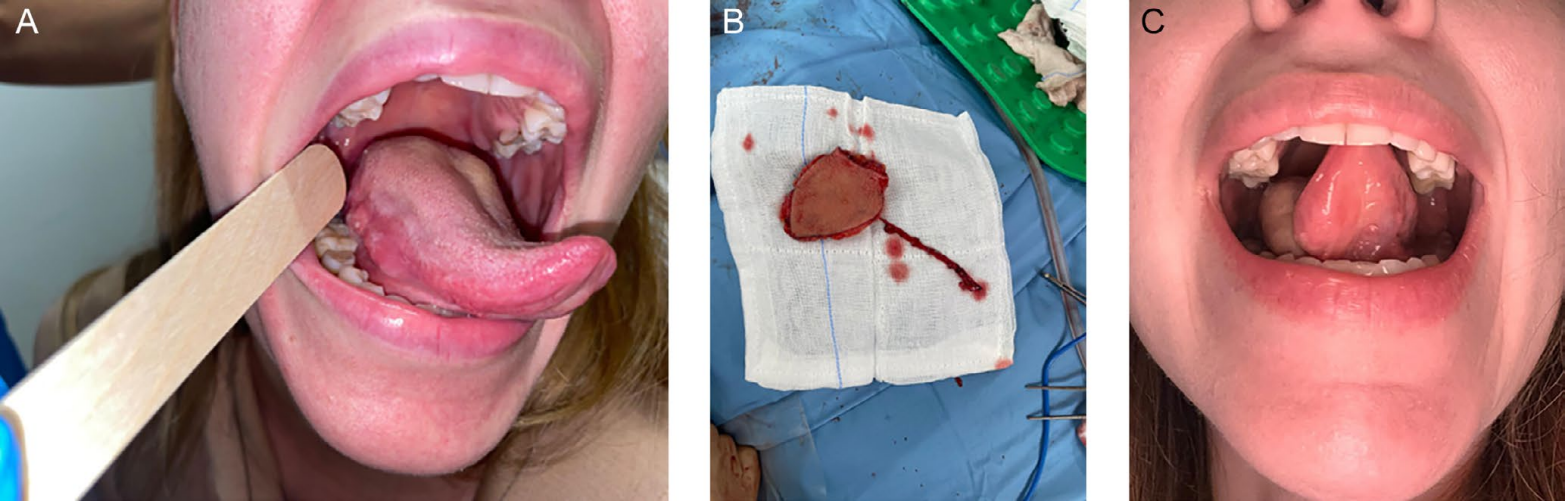
(A) Preoperative view of the tongue tumor. (B) Intraoperative view of the harvested flap. (C) Postoperative view demonstrating tongue mobility.

A 28‐year‐old female patient affected by squamous cell carcinoma arising from the right lateral border of the tongue, staged as pT2 pN1 cM0 (Stage III) Figure 1A. The patient underwent a right compartmental hemiglosso‐pelvectomy associated with a right selective neck dissection (levels I–III). The surgical defect, measuring approximately 4 × 3 cm, was reconstructed using a 5 × 4 cm MSAP flap, with arterial anastomosis to the superior thyroid artery and venous anastomosis to the facial vein Figure 1B. The postoperative course was uneventful: the patient resumed autonomous oral feeding on postoperative day 13 and achieved intelligible speech within 10 days after surgery. At 8‐month follow‐up, the postoperative view confirmed adequate tongue mobility (Figure 1C).

### 2.3 Case 2

**FIGURE 2:**
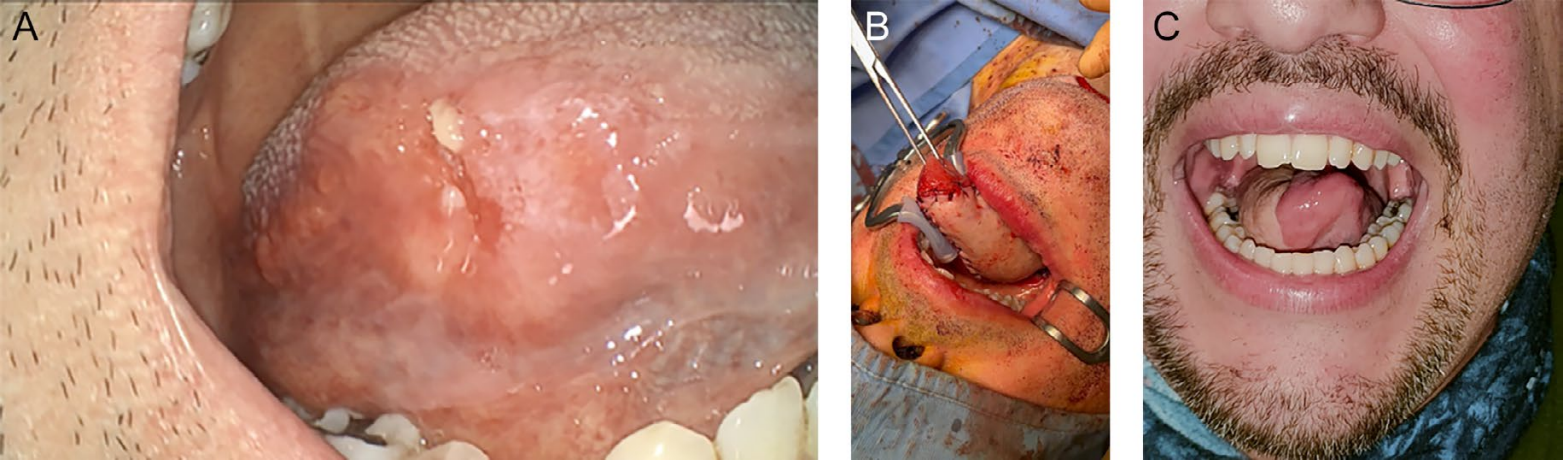
(A) Preoperative view of the tongue tumor. (B) Intraoperative view after flap insetting. (C) Postoperative view demonstrating tongue mobility.

A 50‐year‐old male patient affected by squamous cell carcinoma originating from the right lateral border of the tongue, staged as pT3 pN0 cM0 (Stage III)Figure 2A The patient underwent a right compartmental hemiglosso‐pelvectomy with right selective neck dissection (levels I–IV). The defect, measuring approximately 5 × 4 cm, was reconstructed using a 6 × 5 cm MSAP flap, with arterial anastomosis to the superior thyroid artery and venous anastomosis to the thyrolinguofacial trunk. Figure 2B Oral feeding was resumed on postoperative day 18. Speech intelligibility was satisfactory. At 8‐month follow‐up, the postoperative view confirmed adequate tongue mobility. Figure 2C.

### 2.4 Case 3

**FIGURE 3:**
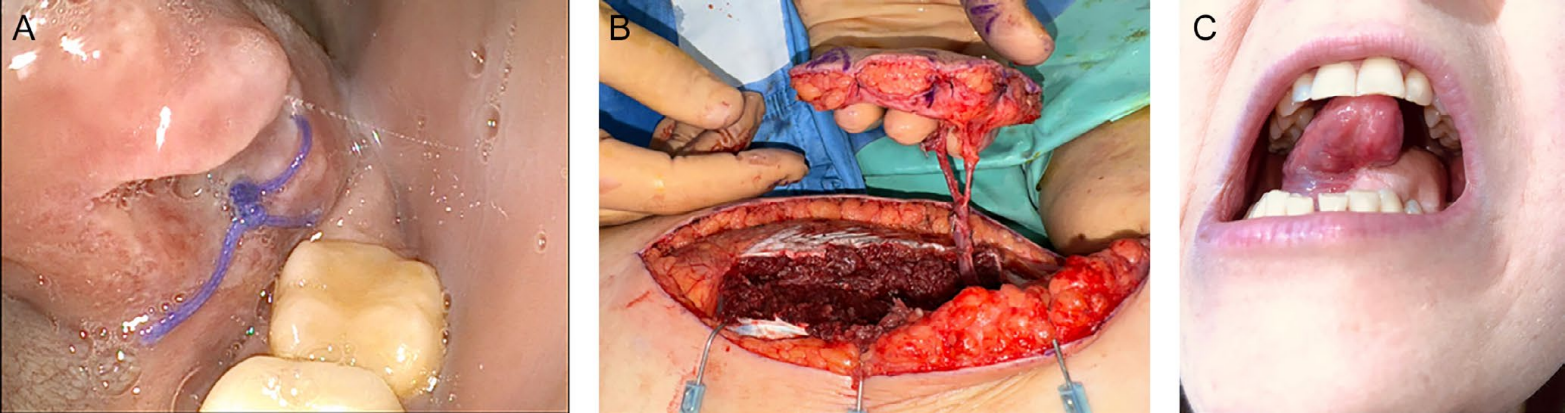
(A) Preoperative view of the tongue tumor. (B) Intraoperative view of the flap harvest. (C) Postoperative view demonstrating tongue mobility.

A 36‐year‐old female patient diagnosed with squamous cell carcinoma of the left lateral border of the tongue, staged as pT2 pN2b cM0 (Stage IVA) Figure 3A. The patient underwent a left compartmental hemiglosso‐pelvectomy associated with bilateral selective neck dissection (levels I–IV). The reconstructive defect, measuring approximately 6 × 5 cm, was repaired with a 7 × 6 cm MSAP flap, with arterial anastomosis to the superior thyroid artery and venous anastomosis to the thyrolinguofacial trunk Figure 3B. The donor site was closed primarily. The postoperative course was uneventful, with resumption of oral feeding on postoperative day 12. At 12‐month follow‐up, the postoperative view confirmed adequate tongue mobility Figure 3C.

## 3 Discussion

Tongue reconstruction after compartmental hemiglossectomy must reconcile three needs: mobility for articulation and bolus control, sufficient bulk for palatal contact, and a supple lining for comfort. Our experience suggests that the MSAP flap provides a pragmatic balance of these features for medium‐sized lateral tongue/floor defects, while limiting donor‐site burden compared with commonly used alternatives such as the RFF flap and the ALT flap (Zhang et al. 2024; Chen et al. 2008). Functionally, all patients in our series achieved early return to oral intake and intelligible speech after rehabilitation, findings that align with prior MSAP outcome reports and head‐to‐head comparisons showing non‐inferiority to RFF and ALT (Al Omran et al. 2023; Hung et al. 2017; Ng et al. 2021). The rationale for our approach also rests on the central role of the tongue tip in consonant production and speech intelligibility; when oncologically feasible, preserving the native tip and leaving it unattached to the flap edge may further enhance residual mobility. To our knowledge, no study has directly compared suturing versus not suturing the preserved tip to the reconstruction.

The tissue characteristics of MSAP help explain these outcomes. MSAP is thinner and more pliable than ALT, avoiding excess bulk that can dampen motion, yet it offers more substance than RFFF, countering the tendency toward late contraction or atrophy observed with very thin reconstructions (Mariniello et al. 2024; Gewirtz et al. 2023). Quantitatively, intraoperative measurements show an average MSAP thickness around 8 mm, intermediate between ALT (~18 mm) and RFFF (~5 mm) (Haq et al. 2025). In our patients, satisfactory intraoral contour was achieved without secondary thinning; where thinning is required, anatomical data support safe suprafascial refinement provided a ~2.5 cm cuff is preserved around perforators (Banjongleelahong et al. 2024).

From a technical perspective, MSAP harvest demands careful planning and intramuscular dissection; pedicle caliber is smaller than with ALT/RFFF and harvest can be time‐consuming. Conversely, MSAP offers consistent pedicle length and caliber suitable for deep cervical recipient vessels and a comfortable inset arc—features valuable in tongue reconstruction workflows (Agrawal et al. 2018). While very large defects may exceed the single‐paddle capacity of MSAP, the dimensions encountered in our series were well within its reconstructive envelope, and all flaps survived uneventfully.

**FIGURE 4:**
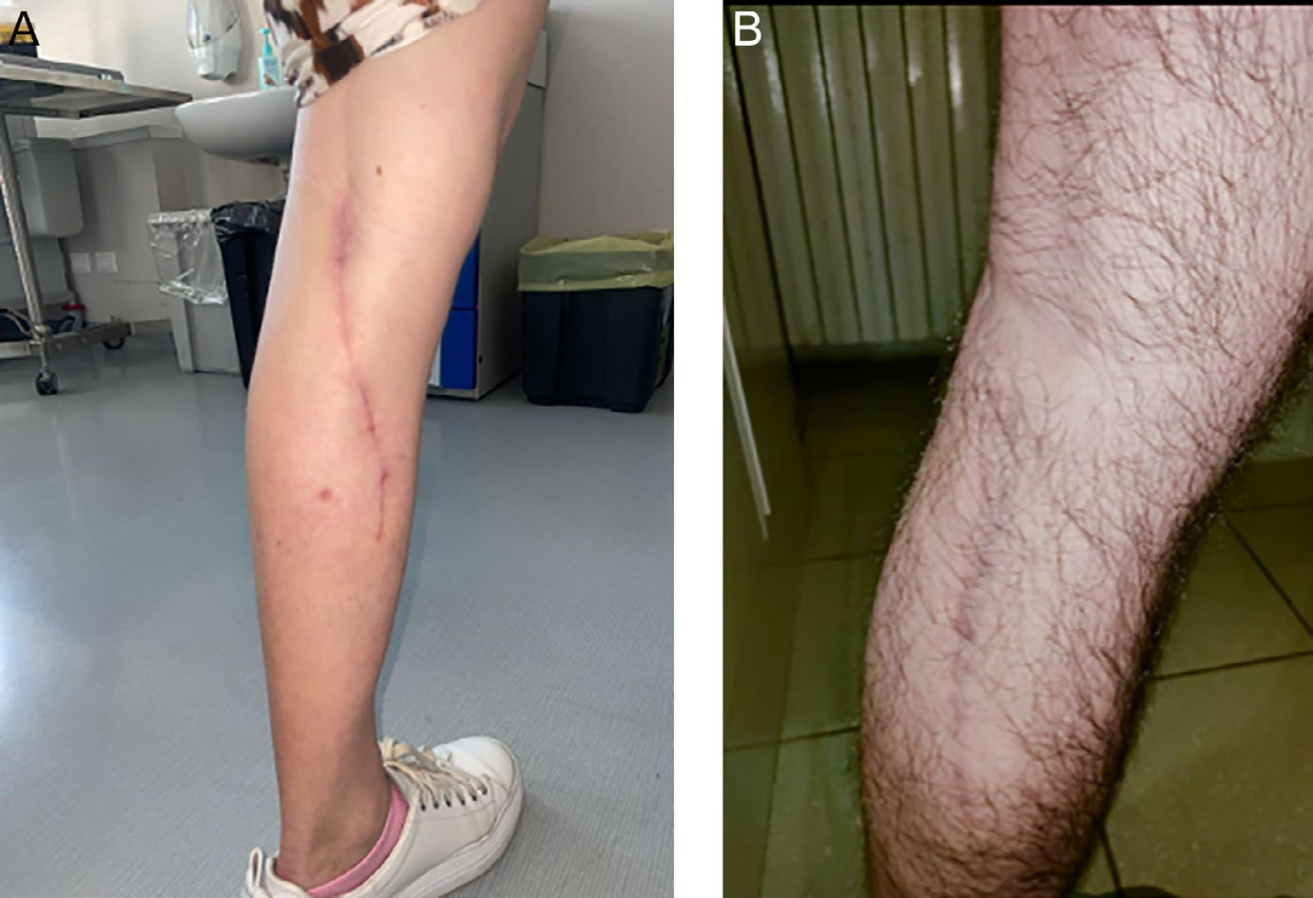
MSAP flap donor‐site scar. (A) Case 3 (female patient). (B) Case 2 (male patient).

Donor‐site morbidity is a decisive factor in flap selection. In our experience the medial‐calf donor site was closed primarily with linear, well‐concealed scars (Figure 4A,B) and no functional deficits, consistent with contemporary series on MSAP in head‐and‐neck reconstruction (Dattilo et al. 2025). Compared with MSAP, the ALT donor site is often closable primarily with low functional sequelae, although grafting increases paresthesia and scar complaints (Collins et al. 2012). The SCIP donor site benefits from a hidden groin‐crease scar but may be prone to lymphorrhea/seroma due to lymphatic density; lymphatic‐sparing harvest can mitigate these risks (Fuse et al. 2022; Iida et al. 2014). RFF flap, while reliable, carries the most visible scar and a well‐documented burden of donor‐arm symptoms despite modern closure strategies (Emanuelli et al. 2023). Within this landscape, we often favor MSAP for the specific lateral tongue/floor subset presented here, while emphasizing that choices should be individualized through patient‐centered counseling.

Regarding SCIP versus MSAP, we recognize SCIP as an excellent option in head‐and‐neck reconstruction for ultra‐thin pliability and a concealed donor site (Mariniello et al. 2024). In our practice, however, MSAP is frequently preferred in the reported defect pattern because it more consistently provides a longer, caliber‐matched pedicle, a stable intermediate thickness that approximates native lingual tissue, and fewer challenges related to perforator variability and lymphatic‐rich fields typical of the groin (Gewirtz et al. 2023; Agrawal et al. 2018; Iida et al. 2014). This is a pragmatic preference rather than a claim of categorical superiority.

Limitations of this work include the small sample size and absence of a control group, which preclude definitive comparative conclusions. Nonetheless, the coherent functional profile observed here, together with published series and comparative analyses, supports MSAP as a middle‐ground solution balancing pliability and volume with low donor‐site cost (Chen et al. 2008; Al Omran et al. 2023; Hung et al. 2017; Ng et al. 2021). Future studies should prospectively evaluate standardized functional endpoints and explicitly test the effect of tongue‐tip preservation without suturing on mobility‐dependent outcomes.

For medium‐sized lateral tongue/floor defects within a compartmental resection pathway, the MSAP flap provides an effective balance of pliability and volume with low donor‐site morbidity; when oncologically feasible, preserving and not suturing the native tongue tip may further enhance mobility and functional recovery.

## Author Contributions

Conceptualization: Francesco Mattioli, Gian Piero Mantovani. Methodology: Gian Piero Mantovani. Data collection: Samuele Baldelli. Writing – original draft: Claudio Gio Francesco Blessent. Writing – review and editing: Massimo Pinelli, Francesco Mattioli.

## Funding

The authors have nothing to report.

## Conflicts of Interest

The authors declare no conflicts of interest.

## Data Availability

The data that support the findings of this study are available from the corresponding author upon reasonable request.
